# Comparative evaluation of Midi Parasep^®^ Solvent Free and the Ritchie concentration technique for helminth and protozoa visualisation in clinical stool samples

**DOI:** 10.1186/s13071-026-07317-0

**Published:** 2026-03-03

**Authors:** Carles Rubio Maturana, Francesc Zarzuela Serrat, Lidia Goterris, Patricia Martínez-Vallejo, Alejandro Mediavilla, Aroa Silgado, Jana Rovira-Plujà, Marc Muixí, Neus Vila-Olmo, Sol María San José-Villar, Nieves Larrosa, Elena Sulleiro

**Affiliations:** 1 Microbiology Department, Vall d’Hebron University Hospital, Vall d’Hebron Research Institute (VHIR), Barcelona, Spain; 2 Department of Microbiology and Genetics, Universitat Autònoma de Barcelona (UAB), Barcelona, Spain; 3 CIBERINFEC, ISCIII-CIBER de Enfermedades Infecciosas, Instituto de Salud Carlos III, Madrid, Spain

**Keywords:** Soil-transmitted helminths, Midi Parasep^®^, Clinical stool samples, Ritchie method, Protozoa, Optical microscopy

## Abstract

**Background:**

This study aimed to evaluate and compare the diagnostic performance of the Midi Parasep^®^ Solvent Free (SF) system and the Ritchie method for detecting helminths and protozoa in clinical stool samples. It also assessed the most suitable concentration technique for different laboratory contexts on the basis of parasitic burden and epidemiological factors.

**Methods:**

A retrospective comparative study was performed with 100 helminth-positive samples from the Drassanes Vall d’Hebron Microbiology Laboratory (Barcelona, Spain). Samples were previously identified using the Ritchie technique and were reprocessed using the Midi Parasep^®^ SF system. All samples were examined by expert microscopists and in accordance with World Health Organization protocols and quality standards. Bivariate analysis was performed using the Z-test or Fisher’s exact test, as appropriate, and differences were considered statistically significant at *P* < 0.05.

**Results:**

The Ritchie method detected 139 parasitic aetiologies, whereas Midi Parasep^®^ SF identified 85, yielding an overall concordance of 61.15%. While protozoan detection showed 100% concordance between both methods, the correlation for helminths was significantly lower (54.6%; *P* < 0.001). Midi Parasep^®^ SF exhibited reduced sensitivity, particularly for larger helminths (e.g., *Strongyloides stercoralis, Schistosoma intercalatum*) and samples with low parasitic burden. In addition, the Alcorfix^TM^ fixative agent caused morphological alterations in some helminth eggs and larvae. Midi Parasep^®^ SF offers operational advantages and hazard reduction; however, the Ritchie method is more sensitive for helminth detection.

**Conclusions:**

The choice of concentration technique should be guided by the clinical context. Midi Parasep^®^ SF is efficient for protozoa and suitable for routine use in high-throughput settings. Conversely, the Ritchie method is preferable when helminth infection is highly suspected, particularly in migrant or travel medicine populations. Moreover, other diagnostic techniques, such as serological assays, could contribute to a more accurate diagnosis, thereby guiding the selection of the most appropriate concentration technique.

**Graphical Abstract:**

**Figure:**
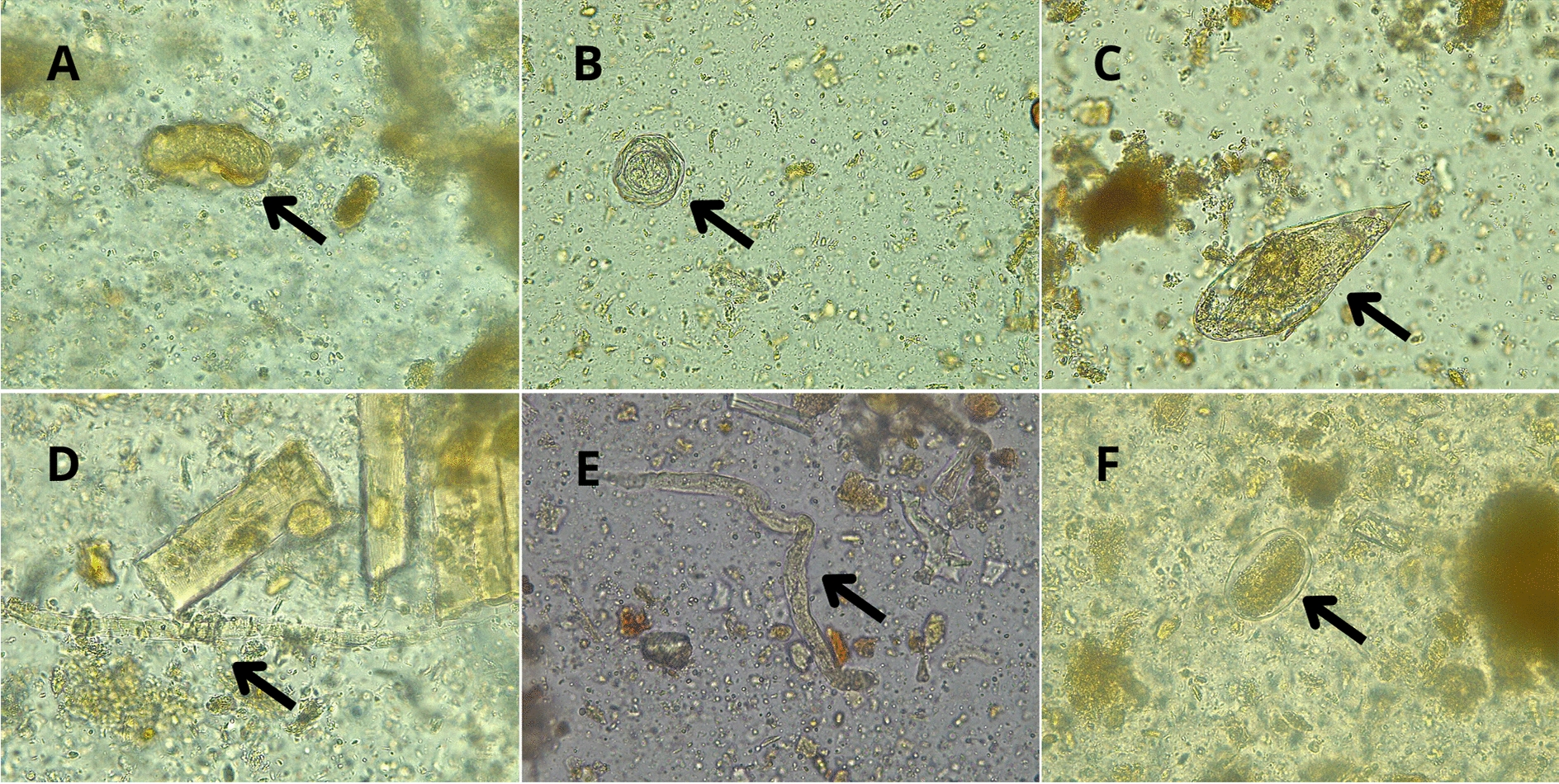
Digital microscopy images of stool wet mount samples with altered helminth morphology after the Midi Parasep^®^ SF Alcorfix^TM^ concentration procedure. All images were acquired at 400 × magnification (10 × ocular lens and 40 × objective lens). **A** Unfertilised *Ascaris lumbricoides* egg; the mammillated outer layer appears twisted or altered. **B** *Hymenolepis nana* egg; the outer membrane, shell, and hexacanth embryo are wrinkled. **C** *Schistosoma intercalatum* egg; the rigid shell is slightly altered, and the miracidium shows abnormalities in the anterior part. **D **and** E** *Strongyloides stercoralis* rhabditiform larvae; cuticle morphology is visibly affected. **F** Hookworm (*Ancylostoma duodenale)* egg; the morula is slightly altered

**Supplementary Information:**

The online version contains supplementary material available at 10.1186/s13071-026-07317-0.

## Background

According to World Health Organization (WHO) data, an estimated 1.5 billion people are infected with soil-transmitted helminths worldwide, posing a significant global health threat [1]. Microscopic examination of faecal samples is the reference standard and recommended technique for the diagnosis of intestinal parasites by the WHO [2]. Several qualitative and quantitative methodologies for coproparasitological diagnosis have been developed; the most common are the Kato–Katz method [3], the Ritchie method [4] and, in recent years, commercial concentrators such as Midi Parasep^®^ [5]. All of these approaches are performance-enhancing techniques, in which faecal material is processed and examined by microscopy. Faecal concentration improves the sensitivity of the technique, and therefore the probability of finding ova, cysts and larvae [6]. The Ritchie method is based on centrifugal sedimentation in a formaldehyde-ether system [7]. Both reagents are considered toxicologically hazardous for environmental and occupational health, resulting in their use being regulated in many European laboratories [8]. The Midi Parasep® Solvent Free (SF) concentration system was specifically developed to eliminate the need for a solvent [6]. It has a small pore size (425 µm), allowing the separation of large faecal particles that might otherwise settle in the cone during centrifugation. In addition, it features a fat dispersion chamber consisting of a secondary filter with a pore size of 220 µm, which retains the smallest faecal debris and separates the fat content from the resulting sediment without adding more solvents [9].

These techniques are useful for visualising protozoa and helminths; however, their morphology and distribution in the sample differ markedly, affecting the technique’s sensitivity.

Helminth species were characterised by their developmental stage, either as eggs or larvae, depending on the organism. *Strongyloides stercoralis* was detected primarily as larvae – reflecting its characteristic life cycle – whereas *Hookworm*, *Ascaris lumbricoides*, *Dicrocoelium dendriticum*, *Diphyllobothrium latum*, *Enterobius vermicularis*, *Hymenolepis nana*, *Schistosoma intercalatum*, *Schistosoma mansoni*, *Taenia* spp. and *Trichuris trichiura* were identified as eggs [10]. Helminth eggs are generally shed intermittently and in variable numbers according to the reproductive cycle of each parasite [11, 12].

Protozoa were defined as trophozoites or cysts, with species-specific considerations. *Entamoeba coli*, *Entamoeba histolytica/dispar*, *Endolimax nana*, *Giardia duodenalis* and *Isospora belli* were detected primarily as cysts or infective forms, whereas *Blastocystis hominis* was identified as vacuolar forms or cysts owing to its unique morphology and controversial life stages [13]. Protozoan forms are generally expelled more uniformly in stool than helminth eggs, reflecting differences in biology and shedding patterns among the studied organisms [14].

Overall, protozoan trophozoites and cysts tend to have a homogeneous distribution in the faeces, whereas helminths are usually heterogeneously distributed, with a low number of eggs per sample, particularly in non-endemic areas [14]. Therefore, some studies determined that serial sampling or observing several sample replicates to improve sensitivity could be a suitable solution for helminth egg visualisation [15, 16]. These scenarios may occur in cases of clinical suspicion of helminths due to eosinophilia [abnormally high eosinophil count (> 500 eosinophils/µL in peripheral blood)] or the patient’s geographical background.

Moreover, other diagnostic methodologies, such as multiplex and syndromic molecular panels for the diagnosis of gastrointestinal infections, are suitable options for detecting multiple parasite aetiologies [17, 18]. Some clinical laboratories have implemented these methodologies for coproparasitological analysis with robust diagnostic results [19–22]. However, other clinical evaluation studies reported poor performance of molecular panels for helminth detection [15, 18]. Moreover, microscopy remains the gold standard, and Polymerase Chain Reaction (PCR) techniques tend to be costly and are often used in combination with conventional microscopy. Serology testing is also commonly employed for screening, and demonstrates high sensitivity for detecting antibodies against intestinal parasites [23–25]. Other alternatives, such as artificial intelligence (AI)-based image microscopy, are currently being developed and validated to automate the gold standard procedure. Detection of protozoa and helminths in stool samples using AI systems has shown promising performance results [26, 27]. Nevertheless, both innovative diagnostics, such as AI-based techniques, and conventional procedures, such as microscopy, still require stool concentration methods to ensure accurate visualisation of the sample. Regardless of the level of automation or image-based analysis, concentration techniques remain a critical pre-analytical step to ensure adequate parasite recovery, morphology preservation, and reliable detection.

In this study, the Midi Parasep^®^ commercial concentrator was evaluated and compared against the Ritchie technique for the detection of helminth and protozoan parasites in clinical stool samples. The main objective of this study was to determine the most appropriate method based on the comparative diagnostic performance for helminth and protozoan parasites.

## Methods

A retrospective comparative study of the Midi Parasep^®^ SF concentrator and the Ritchie method was conducted to evaluate their efficiency in the coproparasitological analysis of helminth infections. The Ritchie method was used in this study because it is the reference-standard concentration technique routinely used in our laboratory protocols. Helminth-positive clinical stool samples were collected and selected from patients attending the International Health and Infectious Diseases Centre, Drassanes Vall d’Hebron (Barcelona, Spain), a multidisciplinary facility providing integrated clinical and laboratory services in infectious diseases, travel medicine, sexual health and community care, with a focus on vulnerable and migrant populations.

Clinical stool samples were previously examined using the Ritchie method in accordance with our laboratory’s standard procedures [28]. All samples were examined by expert microscopists at the International Health and Infectious Diseases Centre, Drassanes Vall d’Hebron, and in accordance with WHO protocols and quality standards [2]. Microscopic identification was based on the Center’s for Disease Control and Prevention (CDC) educational resource concerning the morphology, shape and size of the parasitic form [29]. The Ritchie technique was slightly adapted from the original protocol [20], with laboratory professionals using optical microscopy at 100 × and 400 × magnifications for parasite detection. Approximately 2–3 g of faeces were mixed with 10 mL of 4% formaldehyde and homogenised until a uniform emulsion was formed. The sample was strained through a double-layer gauze into a Falcon® tube, made up to 8 mL with formaldehyde, and 2 mL of diethyl ether was added. The sample was centrifuged at 423 *g* for 3 min, and the supernatant was decanted to allow visualisation of the sediment. One drop of sodium chloride (9 mg/mL) was added to the sample slide to avoid high density and to dilute the preparation.

A total of 100 helminth-positive stool samples identified using the Ritchie technique were concentrated using the Midi Parasep^®^ SF with Alcorfix^™^ (Apacor, Wokingham, UK) 8 mL processing protocol [5]. Moreover, protozoan trophozoites and cysts were also identified when observed, following the same methodology as for helminth visualisation. The concentration method with commercial vials followed Apacor Limited’s protocols [30] and samples were examined by the same laboratory professionals. A small ball of faeces (1 g) was collected with the Midi Parasep^®^ spoon and introduced into the device. The sample was vortexed and centrifuged at 400 *g* for 2 min, and, after decanting the supernatant, the sample was directly observed. All stool samples were visualised with the same optical microscope model (Leica DM750).

Statistical analysis and graph plotting were performed using Microsoft Excel® 2016. For proportion comparisons, bivariate analysis was carried out using the Z-test and Fisher’s exact test as appropriate. Differences were considered statistically significant when the *P*-value was < 0.05.

## Results

Helminth-positive samples identified using the Ritchie concentration technique were employed to evaluate the performance of Midi Parasep^®^ SF. Of these samples, 73/100 (73.0%) were infected by a single parasite and considered mono-infections, while 27/100 (27.0%) were infected by two or more parasites and considered co-infections. Of the 27 co-infections, 18 (66.7%) involved two different parasites, 7 (25.9%) involved three, and one case each (3.7%) involved four or five different parasites. Across all sample types, the per-sample concordance between Midi Parasep^®^ SF and the Ritchie technique was 50/100 (50.0%); samples with any discordant result for a single aetiology were considered non-concordant. Regarding parasite classification, a total of 17 different aetiologies were observed: 11 (64.7%) helminth and 6 (35.3%) protozoan. Parasite aetiologies are specified in Table 1. A total of 139 positive parasite aetiologies were detected in the 100 samples prepared using the Ritchie concentration technique, with *Trichuris trichiura* (25/139, 18.0%) and *Strongyloides stercoralis* (20/139, 14.4%) being the most frequently observed. However, only 85 positive parasite aetiologies were detected in the 100 samples prepared using the Midi Parasep^®^ SF method, with *Trichuris trichiura (*19/85, 22.4%) and *Ascaris lumbricoides* (10/85, 11.8%) being the most represented. Considering the different parasite aetiologies detected, the overall concordance between Midi Parasep^®^ SF and the reference Ritchie method was 85/139 (61.15%). Stratified by type of parasite, there was a 100% (20/20) correlation for protozoan infections, indicating a perfect agreement between both methods for this group. In contrast, for helminth infections, the correlation was 54.62% (65/119). Fisher’s exact test found highly statistically significant differences [*P* < 0.001; odds ratio 34.1 (95% confidence interval: 1.8–635)] in performance for the detection of protozoan and helminth infections. These findings show that the diagnostic techniques had a significantly higher agreement for protozoa than for helminths. All samples with protozoa were co-infections due to the research approach used to identify helminths. The diagnostic identification results from both concentration techniques are presented in Table 1. The proportion test (Z-test) found no statistically significant differences (*P* > 0.05) in the identification of helminths between mono- and co-infection samples prepared with Midi Parasep^®^ SF, nor in any parasite aetiology.

**Table 1:** Comparison of parasite detection rates using Midi Parasep^®^ SF or the Ritchie reference technique, stratified by parasite aetiology and infection type

**Aetiology**	Type of infection	Positive samples using the Ritchie technique (reference method)	Total	Positive using Midi Parasep® SF (%)	Total (%)
**Helminths**					
*Hookworm (E)*	Mono-infections	11	19	5/11 (45.5)	8/19 (42.1)
	Co-infections	8		3/8 (37.5)	
*Ascaris lumbricoides (E)*	Mono-infections	7	13	5/7 (71.4)	10/13 (76.9)
	Co-infections	6		5/6 (83.3)	
*Dicrocoelium dendriticum (E)*	Mono-infections	2	2	0/2 (0)	0/2 (0)
	Co-infections	0		N/A	
*Diphyllobothrium latum (E)*	Mono-infections	1	1	0/1 (0)	0/1 (0)
	Co-infections	0		N/A	
*Enterobius vermicularis (E)*	Mono-infections	3	3	2/3 (66.7)	2/3 (66.7)
	Co-infections	0		N/A	
*Hymenolepis nana (E)*	Mono-infections	8	11	7/8 (87.5)	9/11 (81.8)
	Co-infections	3		2/3 (66.7)	
*Schistosoma intercalatum (E)*	Mono-infections	3	10	0/3 (0)	3/10 (30)
	Co-infections	7		3/7 (42.8)	
*Schistosoma mansoni (E)*	Mono-infections	8	12	4/8 (50)	5/12 (41.6)
	Co-infections	4		1/4 (25)	
*Strongyloides stercoralis (L)*	Mono-infections	16	20	5/16 (31.3)	6/20 (30)
	Co-infections	4		1/4 (25)	
*Taenia* spp.* (E)*	Mono-infections	3	3	3/3 (100)	3/3 (100)
	Co-infections	0		N/A	
*Trichuris trichiura (E)*	Mono-infections	11	25	9/11 (81.2)	19/25 (76)
	Co-infections	14		10/14 (71.4)	
Total helminths	Mono-infections	73	119	40/73 (54.8)	65/119 (54.6)
	Co-infections	46		25/46 (54.3)	
**Protozoa**					
*Blastocystis hominis*	Mono-infections	0	2	N/A	2/2 (100)
	Co-infections	2		2/2 (100)	
*Entamoeba coli (C)*	Mono-infections	0	7	N/A	7/7 (100)
	Co-infections	7		7/7 (100)	
*Entamoeba histo/dispar (C)*	Mono-infections	0	3	N/A	3/3 (100)
	Co-infections	3		3/3 (100)	
*Endolimax nana (C)*	Mono-infections	0	4	N/A	4/4 (100)
	Co-infections	4		4/4 (100)	
*Giardia duodenalis (C)*	Mono-infections	0	3	N/A	3/3 (100)
	Co-infections	3		3/3 (100)	
Isospora belli (C)*	Mono-infections	0	1	N/A	1/1 (100)
	Co-infections	1		1/1 (100)	
Total protozoa	Mono-infections	0	20	N/A	20/20 (100)
	Co-infections	20		20/20 (100)	
Total	Mono-infections	73	139	40/73 (54.8)	85/139 (61.2)
	Co-infections	66		45/66 (68.2)	

SF, Solvent Free; E, egg; L, larvae; spp., species; N/A, not applicable; C, cysts; *oocysts

**Fig. 1:**
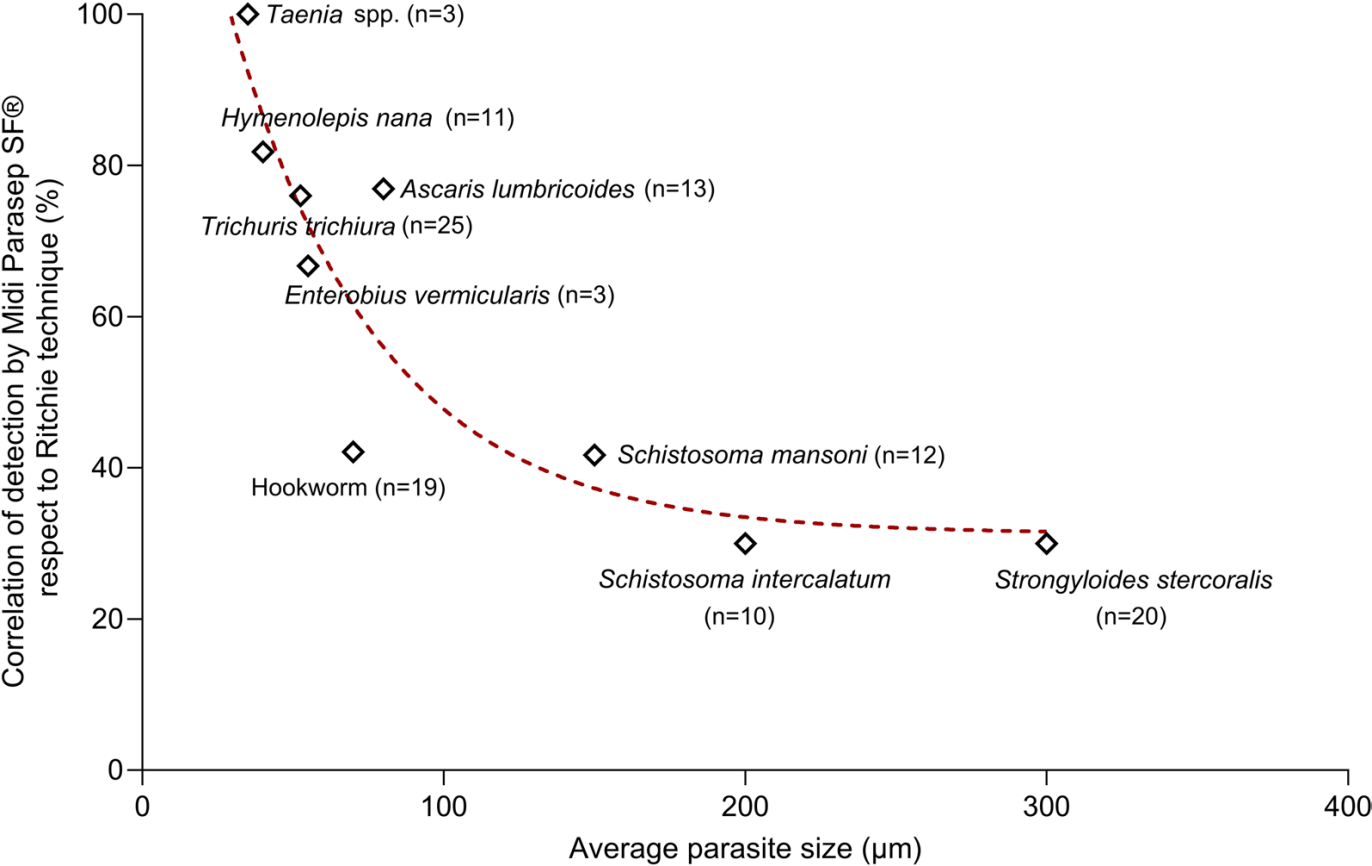
Correlation between average parasite size and detection (%) using Midi Parasep^®^ SF and Ritchie techniques. *n*: number of different samples analysed.

**Fig. 2:**
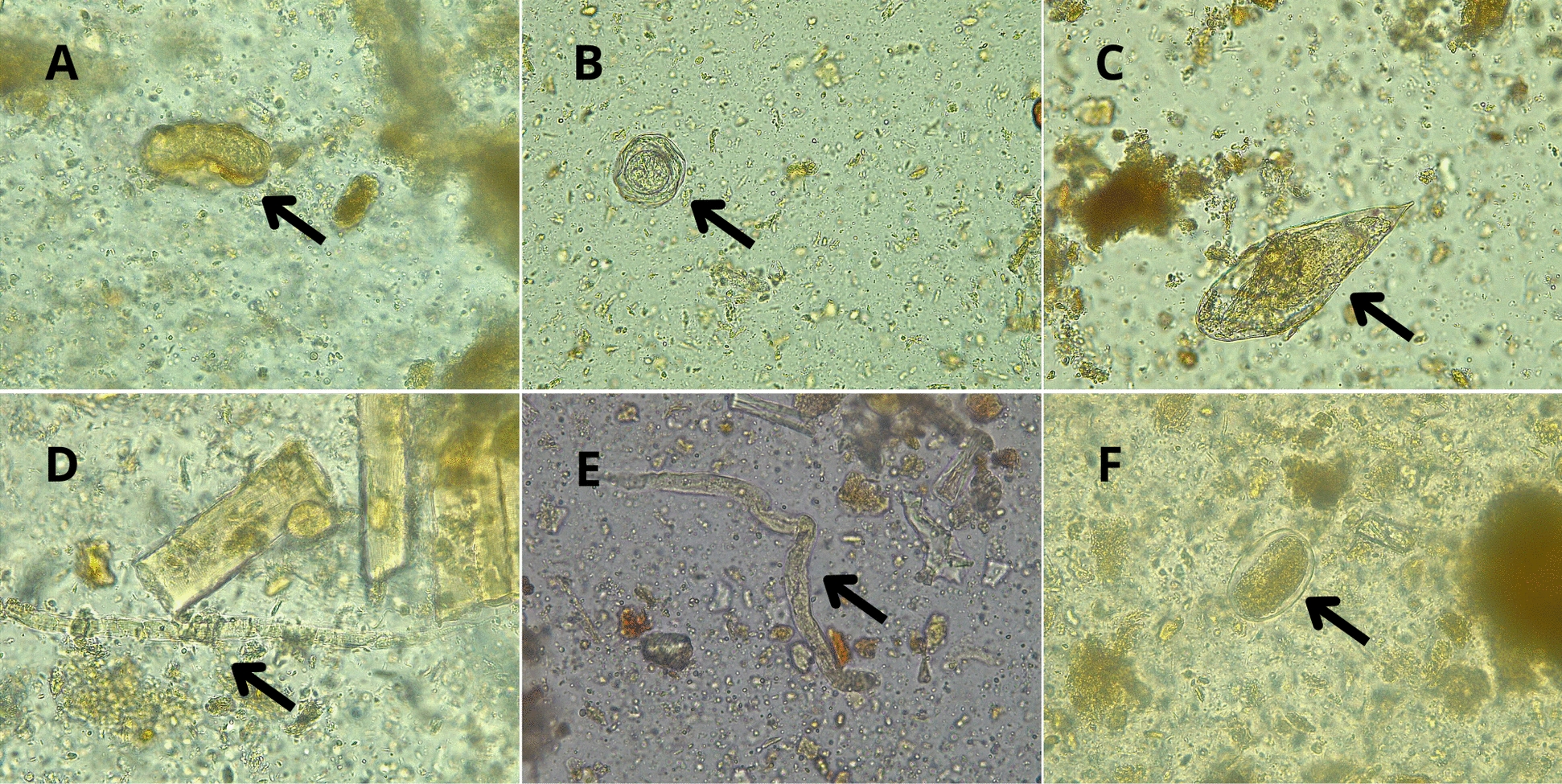
Digital microscopy images of stool wet mount samples with altered helminth morphology after the Midi Parasep^®^ SF Alcorfix^TM^ concentration procedure. All images were acquired at 400 × magnification (10 × ocular lens and 40 × objective lens). **A** Unfertilised *Ascaris lumbricoides* egg; the mammillated outer layer appears twisted or altered. **B** *Hymenolepis nana* egg; the outer membrane, shell, and hexacanth embryo are wrinkled. **C** *Schistosoma intercalatum* egg; the rigid shell is slightly altered, and the miracidium shows abnormalities in the anterior part. **D **and** E** *Strongyloides stercoralis* rhabditiform larvae; cuticle morphology is visibly affected. **F** Hookworm (*Ancylostoma duodenale)* egg; the morula is slightly altered

Diagnostic results show that samples containing larger (length) parasite aetiologies [*Strongyloides stercoralis* (180–380 µm), *Schistosoma mansoni* (114–180 µm) and *Schistosoma intercalatum* (140–240 µm)] have lower correlation values with the Midi Parasep technique. In contrast, the Midi Parasep^®^ concentrator has higher correlation values for samples containing small- to medium-sized parasite aetiologies [*Taenia* spp*.* (30–40 µm), *Trichuris trichiura* (50–55 µm), *Hymenolepis nana* (30–50 µm)]. The relationship between average parasite size and detection correlation using Midi Parasep^®^ is shown in Fig. 1. In addition, with the Midi Parasep^®^ SF system, parasite morphology was slightly affected, potentially leading to microscopic misidentification, thus altering the final diagnosis (Fig. 2 and Supplementary Fig. S1). The Alcorfix^®^ fixative occasionally fails to adequately preserve parasitic forms and may cause morphological alterations in helminth eggs and larvae, as well as protozoan cysts and trophozoites. In contrast, ova and larvae morphologies observed using the Ritchie technique showed no alterations compared with reference forms.

## Discussion

Stool concentration techniques improve parasite visualisation with optical microscopy; however, the method employed is crucial to obtaining better results. In faecal concentration techniques for parasite visualisation, the number of ova, cysts and larvae deposited is affected by the size/volume of the sample employed, the centrifugal force and duration and the presence of a solvent [6]. Moreover, for helminth visualisation, the amount of sample and the egg concentration in faeces are essential for reliable performance. Saez et al. (2011) concluded that, in samples with low parasite count, Midi Parasep^®^ SF could fail to detect parasites, although they did not clarify whether there were differences between helminths and protozoa [6]. In our study, the quantity of sample analysed using the Parasep^®^ method was lower (1 g compared with 2–3 g), in accordance with the manufacturer’s guidelines. This reduced sample amount, together with variations in the proportions of parasitic forms and their heterogeneous distribution, was observed to influence the quality and consistency of microscopic visualisation. Considering these differences, the Ritchie concentration technique yields superior helminth detection results compared with the Midi Parasep^®^ SF method. However, for protozoan visualisation, the two techniques were fully correlated. This may be owing to the lower number of helminth eggs and the different distribution in the sample compared with protozoa. Protozoan cysts tend to have a homogeneous distribution in the sample and a greater number of parasitic forms, whereas helminths usually have a lower presence and their distribution tends to be heterogeneous. In addition, the excretion of helminth eggs tends to be more discontinuous and irregular compared with protozoan cysts, which may further explain the more heterogeneous presence of parasitic forms in stool samples.

Given the pore sizes of the Midi Parasep^®^ SF device (two-stage filtration; 425 µm and 220 µm), this should not be a limiting factor, as most helminth eggs range in size from 30 to 160 µm. However, the results show a lower percentage agreement between the Midi Parasep® and Ritchie techniques when detecting longer helminths. Moreover, considering the helminth samples (*n* > 3), slides containing smaller helminths exhibited a higher percentage agreement between the two concentration techniques. These data suggest that helminth size is crucial for their correct recovery in concentration systems using filters. However, the sample size and the number of parasite eggs and larvae may also influence the final result. *Trichuris trichiura, Taenia* spp., and *Ascaris lumbricoides* infections typically present high parasitic egg loads and significantly higher counts than intestinal schistosomiasis or strongyloidiasis, a fact that could also contribute to the lower concordance between concentration techniques for larger helminths. In the case of *Strongyloides stercoralis* rhabditiform larvae (L1–L2), which measure approximately 180–380 µm [31], they could accidentally become trapped in the pore, a situation accentuated by the low number of larvae usually observed per sample. This limitation may account for the low level of concordance (30%) observed for *S. stercoralis*, linked to the smaller sample size used for Midi Parasep^®^ SF compared with the Ritchie technique. Some studies have posited that higher parasite loads and larger sample volumes for concentration are key determinants for effective detection, rather than parasite size [32, 33].

Centrifugation speed and duration may also affect parasite recovery from clinical stool samples. Manser et al. (2016) demonstrated that the number of parasite stages recovered with the Ridley–Allen concentration method rises with increasing speed and time, without destroying their morphology [34]. Therefore, differences in centrifugation speed and duration in our study may also contribute to the Ritchie technique’s higher sensitivity.

However, Midi Parasep^®^ tubes containing Alcorfix^TM^ provide significant workflow advantages for laboratories processing medium-to-high volumes of samples by consolidating the procedure into a single, enclosed tube [35]. These improvements in workflow and reductions in formalin use in the laboratory compared with the Ritchie technique represent advances in clinical stool sample concentration in high-resource diagnostic parasitology laboratories. Moreover, Alcorfix^TM^ fixative was used to preserve stool samples for parasite detection; however, it may alter the morphology of helminth eggs and larvae as well as protozoa (Fig. 2 and Supplementary Fig. S1), thereby potentially affecting visual assessment by microscopists. These morphological changes may affect diagnostic accuracy. Nevertheless, the fixative is critical for maintaining sample integrity, particularly in high-throughput or delayed-processing laboratory settings, as it serves a role analogous to that of formalin. Taking into account the patient’s origin could also be an important factor in deciding which concentration technique may yield a higher performance. For autochthonous patients (Catalonia/Spain), helminth infections are uncommon; therefore, either technique would be suitable, with Midi Parasep^®^ being simpler to process and offering greater biosafety guarantees. However, for migrant populations, the Ritchie technique would be more suitable for helminth detection owing to its higher sensitivity than Midi Parasep^®^. Moreover, other diagnostic techniques may complement the gold standard, providing appropriate diagnostic guidance. A previous positive serology and/or eosinophilia may suggest the use of the Ritchie concentration technique to detect the presence of helminth eggs and larvae with greater sensitivity. Molecular biology methods, primarily multiplex panels for the detection of several helminth aetiologies, have been introduced in clinical laboratories for diagnostic use. Autier et al*.* (2021) assessed the performance of the Allplex^TM^ GI-Helminth (I) multiplex PCR assay for the detection of *Ancylostoma* spp., *Ascaris* spp., *Enterobius vermicularis*, *Hymenolepis* spp., *Necator americanus*, *Strongyloides* spp., *Taenia* spp. and *Trichuris trichiura* [36]. However, performance results showed a 91–100% concordance between PCR with bead-beating pretreatment and traditional microscopy [36]. Coppens et al. (2024) compared the diagnostic accuracy of the Seegene Allplex^TM^ GI-Parasite and Allplex^TM^ GI-Helminth assays with conventional methods, finding a low correlation (59.1%) for helminth detection [22]. As another example, Robert-Gangneux et al. (2025) evaluated the same Allplex^TM^ molecular panel, which presented optimal performance for the detection of *Giardia intestinalis*, *Cryptosporidium* spp., *Entamoeba histolytica*, *Dientamoeba fragilis* and *Blastocystis* spp. [37]. Nonetheless, the study reported that *Cystoisospora belli* is not targeted by the multiplex assay and that microscopy remains necessary for helminth detection [37]. Therefore, traditional concentration techniques remain essential for detecting intestinal parasites in clinical laboratories, and complementation with other diagnostic techniques could improve the precision of the final diagnosis.

The limitations of our study include the inability to analyse the concentration of parasitic forms – e.g., the number of eggs per slide or per 100 fields – as quantification is not part of routine clinical practice. Moreover, the use of retrospective samples may involve some degree of degradation of parasite form during storage. However, this is unlikely to have influenced the final results, as samples were consistently stored frozen and under controlled conditions. Another limitation is that this is a single-centre study, which may affect the generalisability of the findings to other populations or laboratory settings with different epidemiological contexts or operational workflows.

The comparative evaluation of the performance of Midi Parasep^®^ Solvent Free vials and the Ritchie concentration method demonstrates that both techniques are valuable for detecting intestinal parasites in clinical stool samples, albeit with different strengths and limitations. Midi Parasep^®^ SF achieved complete concordance for protozoan detection, confirming its reliability in this context. However, its sensitivity for helminth detection was markedly lower, particularly in samples with low parasitic burden or larger helminth structures, in which cases, the Ritchie technique showed superior performance. Morphological alterations observed in certain helminth forms following fixation with Alcorfix^TM^ may further compromise diagnostic accuracy.

Despite these limitations, Midi Parasep^®^ SF offers notable operational advantages, including the elimination of formaldehyde use, reduced handling risks and simplified workflow, which render it particularly suitable for high-throughput diagnostic laboratories in high-resource settings. Conversely, in clinical settings with high suspicion of helminth infection, such as among migrant populations, travellers or patients with compatible epidemiological exposure, the Ritchie technique remains preferable owing to its greater diagnostic sensitivity. Complementary diagnostic approaches, including serological and molecular assays, may further enhance detection and provide a comprehensive framework for clinical decision-making.

## Conclusions

Faecal concentration methods remain indispensable for the accurate diagnosis of intestinal parasitic infections. This study demonstrates that while the Midi Parasep^®^ SF system provides operational advantages and efficient detection of protozoa, the Ritchie method maintains superior sensitivity for helminth detection, particularly in samples with low parasite burden. These findings highlight the need for a context-dependent approach to selecting diagnostic methods, considering parasite type, patient epidemiology and laboratory workflow. Optimal diagnostic accuracy may be achieved by implementing adaptive protocols that selectively apply the Midi Parasep^®^ SF or Ritchie method on the basis of these contextual factors. Such protocols would allow laboratories to balance sensitivity, efficiency and resource utilisation, ensuring accurate and timely diagnosis for diverse patient populations. In addition, the superior sensitivity of the Ritchie method for helminth detection has important epidemiological implications. This is particularly relevant in high-risk populations, including migrants, travellers and communities in endemic regions, where undetected infections could perpetuate transmission and morbidity. Future research should focus on validating these context-specific mixed-method approaches, providing evidence-based guidance for laboratories to implement the most effective and efficient diagnostic strategies tailored to their epidemiological and operational settings.

## Supplementary Information

Additional file 1. Figure S1: Digital microscopy images of stool wet mount samples with altered protozoan morphology after the Midi Parasep^®^ SF Alcorfix^TM^ concentration procedure. All images were acquired at 400x magnification with the NanoZoomer S360 Digital Slide Scanner (Hamamatsu Photonics, Japan). **A** *Giardia duodenalis* cyst; the cyst wall is invaginated. **B** *Entamoeba coli* cyst; nuclear morphology is altered. **C** *Entamoeba histolytica / dispar *cyst; the nucleus and endosome were not clearly visible. **D** *Blastocystis hominis*; the vacuole is deformed and undefined **E** *Isospora belli *oocyst*.* **F** *Endolimax nana *cyst

## Data Availability

The data supporting the conclusions of this article are included in this manuscript.

## Abbreviations

AI: Artificial intelligence
CDC: Center’s for Disease Control and Prevention
PCR: Polymerase Chain Reaction
SF: Solvent Free
WHO: World Health Organization
