# Supplementary figures and images for: Comparative evaluation of Midi Parasep® Solvent Free and the Ritchie concentration technique for helminth and protozoa visualisation in clinical stool samples
Source: Parasit Vectors. 2026 Mar 3;19:148. doi: 10.1186/s13071-026-07317-0 (PMC13063889; doi:10.1186/s13071-026-07317-0)

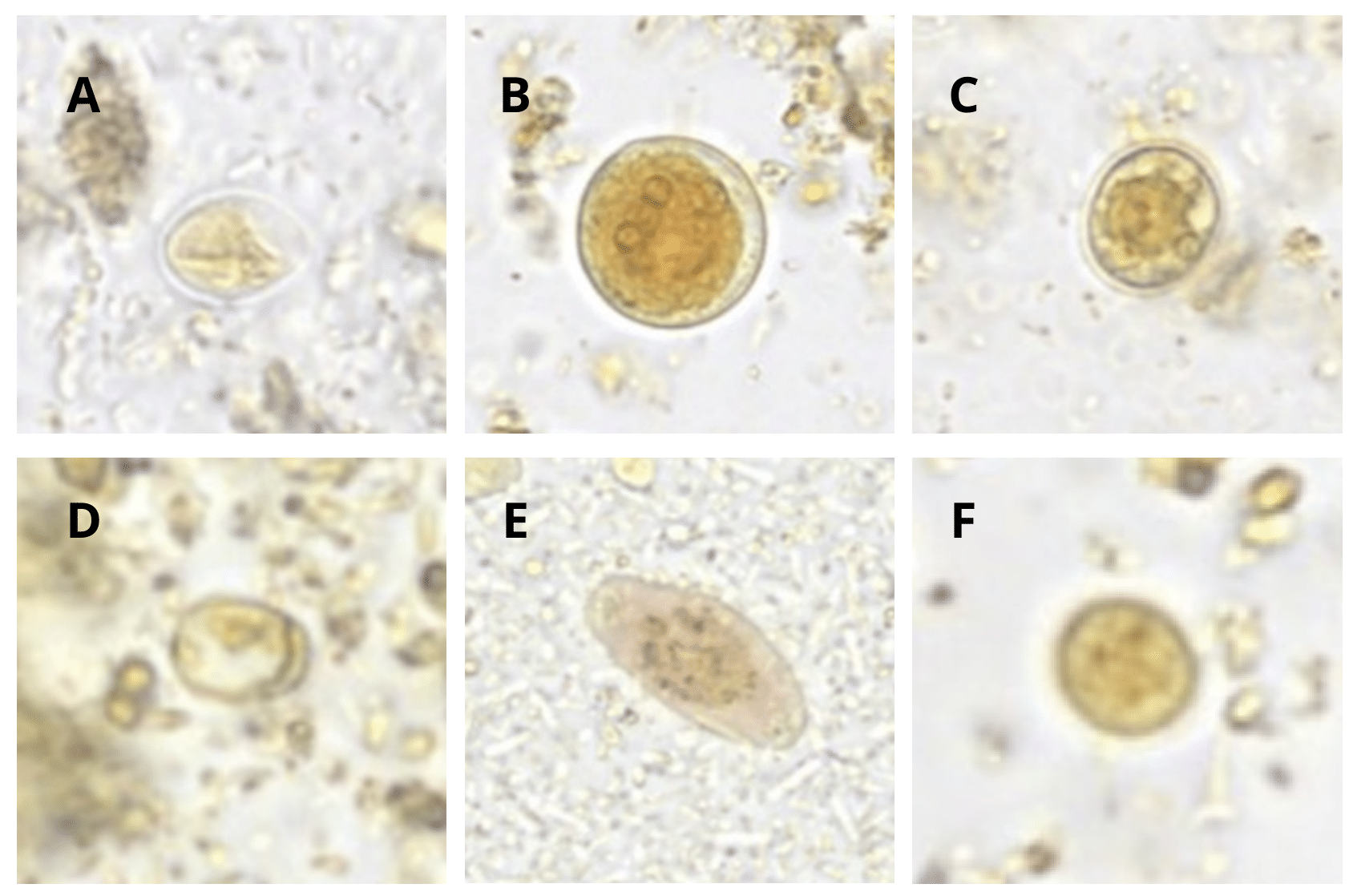

Supplement: Supplementary file 1 — Additional file 1. Figure S1: Digital microscopy images of stool wet mount samples with altered protozoan morphology after the Midi Parasep® SF AlcorfixTM concentration procedure. All images were acquired at 400x magnification with the NanoZoomer S360 Digital Slide Scanner (Hamamatsu Photonics, Japan). A Giardia duodenalis cyst; the cyst wall is invaginated. B Entamoeba coli cyst; nuclear morphology is altered. C Entamoeba histolytica / dispar cyst; the nucleus and endosome were not clearly visible. D Blastocystis hominis; the vacuole is deformed and undefined E Isospora belli oocyst. F Endolimax nana cyst [file 13071_2026_7317_MOESM1_ESM.png]
